# Supplementary material for: End Sequence Analysis Toolkit (ESAT) expands the extractable information from single-cell RNA-seq data
Source: Genome Res. 2016 Oct;26(10):1397–410. doi: 10.1101/gr.207902.116 (PMC5052061; doi:10.1101/gr.207902.116)
Supplement: Supplemental Material [file supp_gr.207902.116_Supp_Materials.tar.gz › ESAT-esat_v0.1/doc/html/class_jama_1_1util_1_1_maths.html]

ESAT: Jama.util.Maths Class Reference


ESAT


- Main Page
- Related Pages
- Packages
- Classes
- Files

- Class List
- Class Index
- Class Hierarchy
- Class Members

All Classes Namespaces Files Functions Variables Enumerator Pages

- Jama
- util
- Maths

Static Public Member Functions |
List of all members

Jama.util.Maths Class Reference

|  |  |
| --- | --- |
| Static Public Member Functions | |
| static double | hypot (double a, double b) |
|  | |

## Member Function Documentation

|  |  |  |  |
| --- | --- | --- | --- |
| static double Jama.util.Maths.hypot | ( | double | *a*, |
|  |  | double | *b* |
|  | ) |  |  |

static

sqrt(a^2 + b^2) without under/overflow.

---

The documentation for this class was generated from the following file:

- java/Jama/util/Maths.java


---

Generated on Fri Aug 15 2014 15:11:06 for ESAT by  

 1.8.7
